# A Novel Genetic Screen Identifies Modifiers of Age-Dependent Amyloid β Toxicity in the *Drosophila* Brain

**DOI:** 10.3389/fnagi.2017.00061

**Published:** 2017-03-14

**Authors:** Lautaro F. Belfiori-Carrasco, María S. Marcora, Nadia I. Bocai, M. Fernanda Ceriani, Laura Morelli, Eduardo M. Castaño

**Affiliations:** ^1^Laboratorio de Amiloidosis y Neurodegeneración, Fundación Instituto Leloir-Instituto de Investigaciones Bioquímicas de Buenos Aires, Consejo Nacional de Investigaciones Científicas y Técnicas (CONICET)Buenos Aires, Argentina; ^2^Laboratorio de Genética del Comportamiento, Fundación Instituto Leloir-Instituto de Investigaciones Bioquímicas de Buenos Aires, Consejo Nacional de Investigaciones Científicas y Técnicas (CONICET)Buenos Aires, Argentina

**Keywords:** amyloid β, Alzheimer’s disease, neurodegeneration, genetic screen, *Drosophila*, dementia

## Abstract

The accumulation of amyloid β peptide (Aβ) in the brain of Alzheimer’s disease (AD) patients begins many years before clinical onset. Such process has been proposed to be pathogenic through the toxicity of Aβ soluble oligomers leading to synaptic dysfunction, phospho-tau aggregation and neuronal loss. Yet, a massive accumulation of Aβ can be found in approximately 30% of aged individuals with preserved cognitive function. Therefore, within the frame of the “amyloid hypothesis”, compensatory mechanisms and/or additional neurotoxic or protective factors need to be considered and investigated. Here we describe a modifier genetic screen in *Drosophila* designed to identify genes that modulate toxicity of Aβ42 in the CNS. The expression of Aβ42 led to its accumulation in the brain and a moderate impairment of negative geotaxis at 18 days post-eclosion (d.p.e) as compared with genetic or parental controls. These flies were mated with a collection of lines carrying chromosomal deletions and negative geotaxis was assessed at 5 and 18 d.p.e. Our screen is the first to take into account all of the following features, relevant to sporadic AD: (1) pan-neuronal expression of wild-type Aβ42; (2) a quantifiable complex behavior; (3) Aβ neurotoxicity associated with progressive accumulation of the peptide; and (4) improvement or worsening of climbing ability only evident in aged animals. One hundred and ninety-nine deficiency (Df) lines accounting for ~6300 genes were analyzed. Six lines, including the deletion of 52 *Drosophila* genes with human orthologs, significantly modified Aβ42 neurotoxicity in 18-day-old flies. So far, we have validated *CG11796* and identified *CG17249* as a strong candidate (whose human orthologs are *HPD* and *PRCC*, respectively) by using RNAi or mutant hemizygous lines. *PRCC* encodes proline-rich protein PRCC (ppPRCC) of unknown function associated with papillary renal cell carcinoma. *HPD* encodes 4-hydroxyphenylpyruvate dioxygenase (HPPD), a key enzyme in tyrosine degradation whose Df causes autosomal recessive Tyrosinemia type 3, characterized by mental retardation. Interestingly, lines with a partial Df of *HPD* ortholog showed increased intraneuronal accumulation of Aβ42 that coincided with geotaxis impairment. These previously undetected modifiers of Aβ42 neurotoxicity in *Drosophila* warrant further study to validate their possible role and significance in the pathogenesis of sporadic AD.

## Introduction

Alzheimer’s disease (AD) is the most prevalent form of dementia in the aged population worldwide and its impact is steadily growing due to the extension of life expectancy (Cacace et al., 2016; Scheltens et al., 2016). More than 95% of AD cases are sporadic, with age and the epsilon 4 allele of the apolipoprotein E gene as the major risk factors. Rare familial forms are associated with mutations in the amyloid precursor protein and presenilin 1–2 genes (Campion et al., 1995; Newman et al., 2007; Kandimalla et al., 2011, 2012; De Strooper and Karran, 2016).

AD brain is characterized by a pervasive synaptic loss and the accumulation of protein aggregates mostly composed of Aβ42 and microtubule-associated protein tau. Oligomeric species of Aβ42 have been proposed as early pathogenic molecules by inducing mitochondrial and endoplasmic reticulum stress, an increase in reactive oxygen species formation and action potential abnormalities (Karran et al., 2011; De Strooper and Karran, 2016). AD tau is excessively phosphorylated and aggregates intracellularly leading to microtubule instability and organelle failure (Khan and Bloom, 2016). However, the accumulation of Aβ and phospho-tau is not sufficient for the development of AD. Large autopsy series show that about 30%–40% of individuals can sustain a normal or nearly normal cognitive function at a very old age despite extensive Aβ and phospho-tau pathology (Bennett et al., 2006; Maarouf et al., 2011; Perez-Nievas et al., 2013). Several hypothesis have been put forward to explain such clinico-pathological dissociation, including differences in “cognitive/brain reserve” or the presence of compensatory mechanisms at a functional or molecular level (Maarouf et al., 2011; Steffener and Stern, 2012). In this context, the search for novel genetic and epigenetic factors that partake in neurotoxicity mechanisms related to Aβ is of key importance for understanding the disease process.

*Drosophila* is widely used for genetic screens applied to study the molecular bases of neurodegenerative disorders including AD (Crowther et al., 2005; Moloney et al., 2010; Lenz et al., 2013; Prüßing et al., 2013; Shulman et al., 2014; Fernandez-Funez et al., 2015; Liu et al., 2015). Major advantages of this animal model include a complex CNS, the fact that about 70% of human genetic diseases have a *Drosophila* genetic counterpart (Jackson, 2008; Bouleau and Tricoire, 2015; Lim et al., 2016) and the availability of large collections of mutant and transgenic lines.

Forward genetic screens in *Drosophila* have been used to identify modifiers of Aβ neurotoxicity. Cao et al. (2008) used a collection of transgenic lines carrying directionally inserted P elements and screened for enhancers or suppressors of a rough eye phenotype induced by Aβ42. In this way, they identified candidate genes involved in cellular processes such as transcription regulation, proteolysis in the secretory pathway and cholesterol metabolism (Finelli et al., 2004; Cao et al., 2008). By screening a collection of chromosomal deletions, the same group found that the toll-NFκB pathway enhanced both Aβ-induced rough eye and a negative effect upon life span (Tan et al., 2008). Rival et al. (2009) screened 3000 lines carrying P element inserts for modifiers of a shorter life span induced by the “Arctic” variant of Aβ42 (AβE22G) associated with familial AD. Notably, they found that genes associated with redox or antioxidant activities were strong modifiers of AβE22G neurotoxicity (Rival et al., 2009). By inducing misexpression of genes involved in specific developmental pathways, several modifiers of Aβ42 toxicity upon photoreceptors have been described (Moran et al., 2013). In addition to the eye phenotype and life span, the gravitaxis behavior (negative geotaxis) can be used for genetic screening. This test provides easily quantifiable data, explores a complex behavior of the *Drosophila* CNS and allows a rapid assessment of age-dependent Aβ toxicity. Recently, Liu et al. (2015) developed an automatic device for the Rapid Iterative Negative Geotaxis (RING) assay and screened a collection of chromosomal deletions to find modifiers of AβE22G neurotoxicity upon the giant fiber system neurons (Gargano et al., 2005; Liu et al., 2015).

The aim of the present study was to develop a modifier screen designed to study the effect of chromosomal deletions upon neuronal toxicity mediated by pan-neural expression of wild-type Aβ42 in the CNS (the major isoform that accumulates in the brain of sporadic AD patients). Fly lines with defined genomic deletions were found to exert a dominant effect under the presence of Aβ42. Deficiency (Df) lines that significantly enhanced age-dependent Aβ42 toxicity included *CG17249* and *CG11796* whose human orthologs are *PRCC* and *HPD*, respectively. *PRCC* encodes proline-rich protein PRCC (ppPRCC), a protein of unknown function associated with renal cell carcinomas. *HPD* encodes 4-hydroxy-phenylpyruvate dioxygenase (HPPD), a key enzyme in tyrosine degradation.

## Materials and Methods

### Fly Stocks

Flies were raised at 25°C in a standard corn meal with a light:dark cycle of 12 h:12 h. The line expressing Aβ1-42 fused with the rat pre-proenkephalin signal peptide was kindly provided by Dr. Mary Konsolaki (Rutgers University). The upstream activating sequence (UAS)-Aβ42 construct is inserted in the 2nd chromosome. Lines *w*^1118^ #5905 (+), *elav*^c155^ [Gal4] #458 (G4), lines from the Df kit and the mutants for *CG11796* #51528 and *CG17249* #16098 were obtained from Bloomington *Drosophila* Stock Center (NIH P0OD018537). The CG11796 RNAi line #103482 was obtained from VDRC Stock Center. The *elav* [Gal4]; [UAS] Aβ42/Cyo line (G4 > Aβ42) was generated for the screen.

### RING Assay

Groups of 30–40 male flies were raised at 25°C in 4-inch glass vials with food replacement every 2–3 days. The geotaxis behavior was tested using the RING assay as described (Gargano et al., 2005). The day before the test, 10 flies were shortly anesthetized with CO_2_ and placed into a fresh vial. They were let to recover overnight at 25°C, transferred to clear glass vials and placed them in the negative geotaxis device. The device was tapped three times in rapid succession to initiate the response and climbing was recorded for 10 s. The climbed distance in cm was measured for each fly and the average height from five technical replicates per genotype was calculated using the Scion Image software.

### SDS-PAGE and Western Blots

Forty heads from 5 to 18-day-old flies were homogenized in 60 μl of RIPA buffer, pH 7.4, containing 1% SDS, 5 mM EDTA, 5 mM EGTA, 1 mM PMSF, 0.5 μg/ml leupeptin, 0.5 μg/ml aprotinin, 1 mg/ml pepstatin and 50 mM NaF. Homogenates were centrifuged at 10,000× g for 1 h at 4°C. Twenty μl of the supernatant containing ~150 μg of total proteins, were resolved by sodium dodecyl sulfate-polyacrylamide gel electrophoresis (SDS-PAGE) in a 12.5% Tris-tricine gel. After transfer to polyvinylidene fluoride membranes, proteins were analyzed by Western blot. Aβ42 was detected with anti-Aβ monoclonal 6E10 (Biolegend Co.) used at 1:1000. Actin was detected with rabbit polyclonal anti-actin (Sigma) at 1:1000. After washing with PBS-T, membranes were incubated anti-rabbit or anti-mouse horseradish peroxidase-labeled IgGs (Dako, Denmark) at 1:10000. Immunoreactivity was visualized by chemiluminescence with ECL Prime (GE Bioscience, Piscataway, NJ, USA) and scanned with an Image Quant LAS 4000 apparatus (GE Bioscience, Piscataway, NJ, USA). For relative quantitation, optical densities from each lane were obtained and analyzed with the ImageJ software. Synthetic Aβ1-42 was obtained from American Peptide Co.

### Inmunohistochemistry and Thioflavin S Staining

Adult heads were fixed with 4% paraformaldehyde in phosphate buffered saline (PBS) for 45 min at room temperature (RT). Fly brains were dissected in PBS containing 0.1% Triton X-100 (PT). Brains were blocked in 10% normal goat serum for 1 h in PT and incubated with antibody 6E10 at 4°C overnight. After incubation with Cy3-labeled anti-mouse antibody (Jackson InmunoResearch, West Grove, PA, USA) for 2 h at RT, brain tissue was stained with DAPI, washed with PBS and mounted in PBS containing 80% glycerol. For amyloid fibril staining, brains were incubated in 50% ethanol containing 1% thioflavine S (ThS; Sigma, St.Louis, MO, USA) overnight at 4°C. Samples were washed with PBS containing 50% ethanol and mounted in 80% glycerol. Brain samples from a transgenic mouse carrying the “Swedish” mutation of amyloid precursor protein (Tg2576) were used as positive controls. Images were captured with a Zeiss LSM 510 Meta Confocal microscope.

### Histology and Vacuolization Assessment

Fly heads were fixed overnight in Carnoy solution (60% ethanol, 30% chloroform, 10% acetic acid) at 4°C and dehydrated in increasing concentrations of ethanol. Then, they were treated with butanol:ethanol (1:1), butanol:toluene (1:1) and toluene 30 min each, and finally soaked in toluene:paraffin (1:1) for 30 min at 65°C. After a 2-h incubation at 65°C in pure paraffin, heads were embedded and cut in 8 μm serial frontal sections. After H&E staining, images were captured using an OLYMPUS B × 50 Microscope and analyzed with the ImageJ software. Brain tissue loss was quantified as described (Sarantseva et al., 2009). The area occupied by vacuoles with a diameter of at least 3 μm was divided by the total area of the section and expressed as percentage of area loss. At least eight brains per genotype were analyzed.

### Genetic Screen

**Figure 1:**
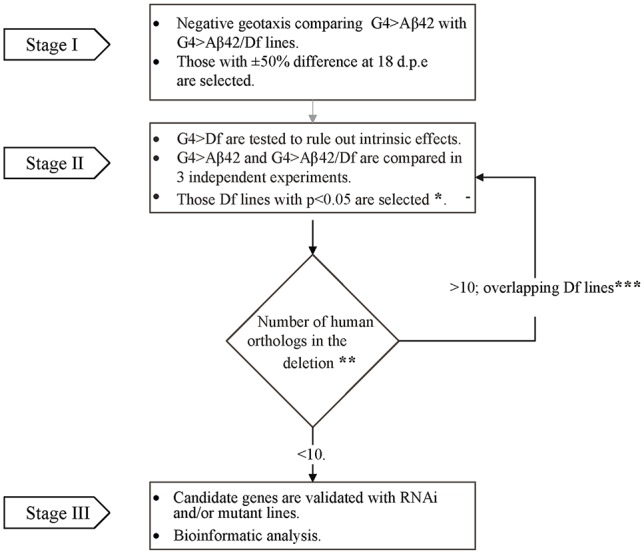
**Flow chart illustrating the overall strategy and steps of the modifier genetic screen.** In stage I, G4 > amyloid β peptide 1-42 (Aβ42) line was compared to each of the G4 > Aβ42/Deficiency (Df) lines in a single negative geotaxis experiment. *In stage II, those G4 > Aβ/Df lines selected in stage I were examined in three independent biological experiments for statistical significance at 18 days post eclosion (d.p.e), (one-way ANOVA followed by least significant difference (LSD) Fisher’s test *p* < 0.05). **Human orthologs were defined as those with the highest score according to *Drosophila* RNAi Screen Center (DRSC) integrative ortholog prediction tool (DIOPT). Depending on the number of deleted orthologs (> or ≤ 10), Df lines were selected for stage III or back to stage II analysis with overlapping deletions to narrow down the number of candidates (***Overlapping deletions were compared in three independent biological experiments).

To perform the genetic screen, the G4 > Aβ42 line was mated with Df lines from the Bloomington Df kit (Cook et al., 2012; Cook, 2016) to generate *elav*^c155^ [Gal4]; [UAS] Aβ42/+> Df/+ (G4 > Aβ42/Df). The experimental design consisted of three stages (Figure 1). In stage I, G4 > Aβ42/Df lines were analyzed at 5 and 18 days post-eclosion (d.p.e) to find a modified phenotype as compared to G4 > Aβ42. Genetic controls included G4>+ and +>Aβ42. Those Df lines that showed a difference of at least 50% in negative geotaxis only at 18 d.p.e in a single biological experiment were selected. In stage II, each chromosomal deletion; *elav*^c155^ [Gal4]; Df/+ (G4 > Df) was assessed to rule out that it did not affect negative geotaxis in the absence of Aβ42 expression. Three independent biological experiments were performed comparing G4 > Aβ42 with G4 > Aβ42/Df to select the Df lines that reached statistical significance. Deleted genes were queried for the identification of human orthologs with expression in the adult CNS (see below). If the deletion was large and included more than 10 human orthologs, overlapping deletions were analyzed as in stage II to reduce the number of candidates. Deletions with less than 10 human orthologs were selected for analysis with RNAi or mutant lines in stage III.

### Bioinformatic Analysis of Deficiency Lines

Genomic deletions were queried in Bloomington Stock web page^1^. The corresponding gene list was obtained from FlyBase^2^ (Attrill et al., 2016) using the GBrowse function and the Hit List tool. Each gene was searched for its human ortholog with the highest weighted score using the *Drosophila* RNAi Screen Center (DRSC) Integrative Ortholog Prediction Tool (DIOPT) from the DRSC^3^. Fly gene expression was searched in NCBI web page^4^ and the RNA-seq Profile provided by FlyBase on the gene query subtitle expression data^5^. Gene products, their known functions, patterns of expression in humans, protein-protein interactions and association with human diseases were obtained from UNIPROT^6^, Genecards^7^ and OMIM^8^ databases.

### Preparation of cDNA Samples and Quantitative Real-Time PCR

RNA from 35 fly heads was extracted with the TriZol reagent (Invitrogen) according to manufacturer’s instructions. cDNA was generated from 3 μg of RNA, previously treated with DNAse (Promega) using the SuperScript III system (Invitrogen). SYBR-Green quantitative real-time PCR (qRT-PCR) was performed using KAPA SYBR_FAST Universal 2X qPCR Master Mix. Reactions were run in a Stratagene Mx3005P cycler (Agilent Technologies) and analyzed by the calibration curve method. For *CG11796* primers 5′AAAGGAACCAAACCTGAA GC 3′ (forward) and 5′ATCCCTGATAGCCAAGTGGT 3′ (reverse) were used. *RPL32* was amplified for normalization using the following primers: 5′ATGCTAAGCTGTCGCACA AATG 3′ (forward) and 5′GTTCGATCCGTAACCGATGT 3′ (reverse).

### Statistical Analysis

Results are presented as the mean ± SEM of at least three independent biological experiments unless otherwise stated. Data were analyzed by repeated measures (RM) two-way ANOVA with *post hoc* Bonferroni’s test, RM one-way ANOVA followed by Least Significant Difference (LSD) Fisher’s test or Student’s *t* test using the Prism^®^ Graphpad 6 software. Wilcoxon non-parametric test were used when indicated. The level of significance was set at *p* < 0.05.

## Results

### G4 > Aβ42 Line Shows a Moderate and Age-Dependent Toxic Phenotype

**Figure 2:**
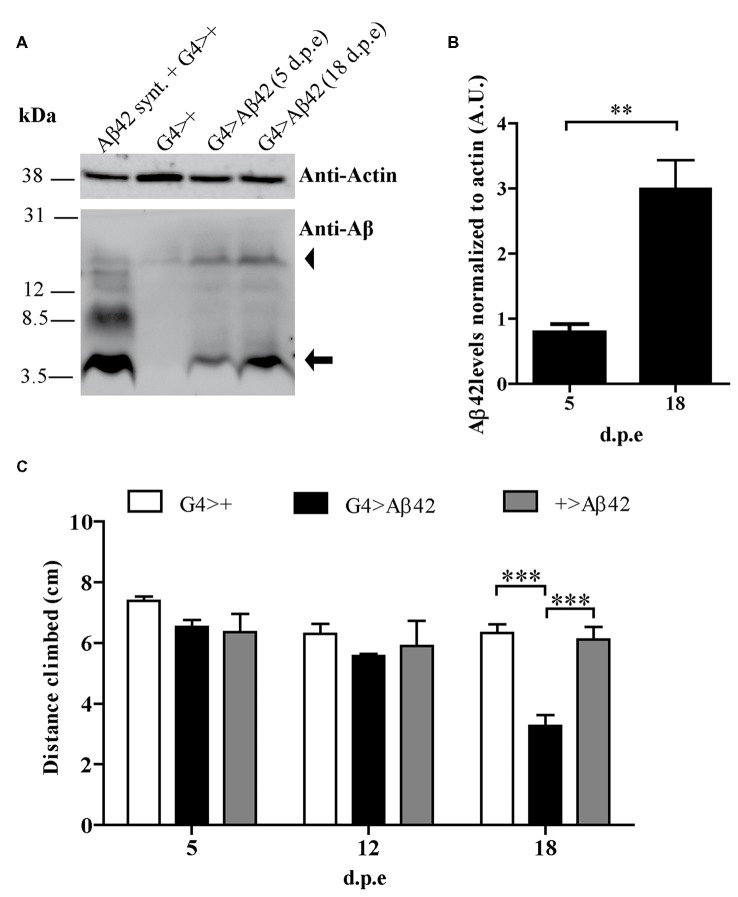
**(A)** Representative Western blot of fly brain homogenates at 5 and 18 d.p.e showing Aβ42 expression detected with anti-Aβ monoclonal antibody 6E10. The 4.5 kDa band (arrow) indicates Aβ42 correctly processed in the secretory pathway. The arrowhead indicates a band consistent with sodium dodecyl sulfate (SDS)-resistant Aβ42 oligomers. A G4>+ brain homogenate was spiked with synthetic Aβ1-42 (Aβ42 Synt.) for electrophoretic mobility control. Membrane was cut above the 31 kDa marker and probed with anti-actin for normalization. **(B)** Quantification of Aβ42 levels relative to actin in arbitrary units (A.U.). Bars represent the mean ± SEM from three independent experiments; **p < 0.01 (Student’s *t*-test). **(C)** Pan-neuronal Aβ42-expressing flies (G4 > Aβ42) showed climbing impairment at 18 d.p.e as compared with genetic controls (G4>+ and +>Aβ42). Bars represent the mean ± SEM from at least three independent biological experiments; ***p < 0.001 (repeated measures [RM] two-way ANOVA followed by Bonferroni’s *post hoc* test).

A transgenic line with constitutive, pan-neuronal expression of Aβ42 maintained at 25°C was examined as a candidate for the screen. Western blots of fly head homogenates showed a ~4.5 kDa band consistent with detergent-soluble Aβ42 correctly targeted and cleaved in the secretory pathway. A minor band consistent with SDS-resistant Aβ42 oligomers was also seen. Between 5 and 18 d.p.e there was a robust 3-fold increase of Aβ42 levels (Figures 2A,B). Negative geotaxis was not impaired in 5-day-old flies as compared with controls, strongly suggesting that there were no developmental effects upon the CNS due to Aβ42 expression. In 18-day-old flies, a significant decrease in climbing ability (~50%) was apparent only in Aβ42-expressing animals as compared to genetic controls, G4>+ and +>Aβ42 (Figure 2C). Microscopic examination of the brains of affected flies revealed very mild vacuolization and negative ThS staining (see below). Therefore, this line showed age-dependent Aβ42 accumulation and CNS neurotoxicity, and the magnitude of the functional decline was optimal for the search of enhancers and suppressors. In addition, the accretion of non-fibrillar Aβ42 suggests that toxicity was induced by soluble oligomers, as proposed for AD. Taken together, these features and experimental conditions made this Aβ42 transgenic line highly suitable for a forward genetic screen.

### Identification of Df Lines that Modify Age-Dependent Aβ42 Toxicity

**Figure 3:**
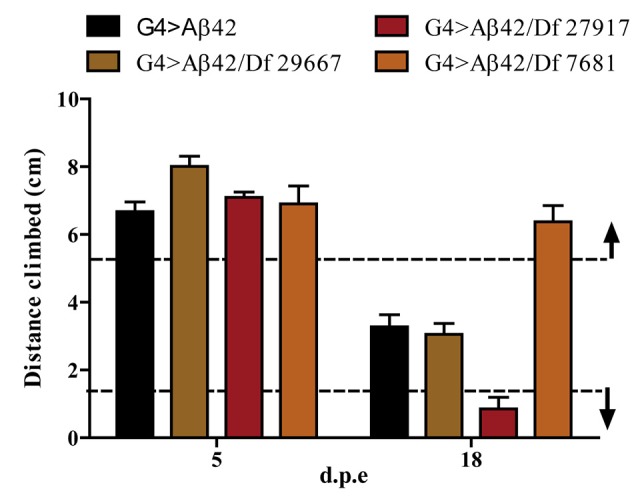
**Negative geotaxis assay of G4 > Aβ42 compared to G4 > Aβ42/Df at 5 and 18 d.p.e.** The graphic shows examples of the three possible outcomes according to the quantitative criterion of at least a 50% difference in negative geotaxis (dashed lines): Df 29667 had no modifier effect, Df 27917 worsened and Df 7681 improved the climbing ability of Aβ42-expressing flies at 18 d.p.e. Bars represent the mean ± SEM from a single biological experiment (five technical repeats) and therefore, at this stage of the screen, no statistical analyses were performed.

**Figure 4:**
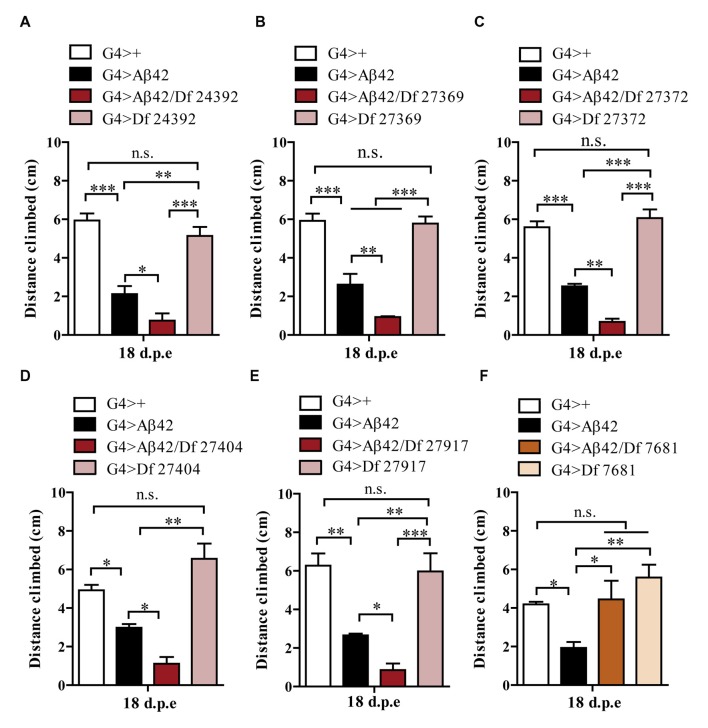
**Negative geotaxis assay of G4>+, G4 > Aβ42, G4 > Aβ42/Df and G4 > Df at 18 d.p.e. (A)** Df 24392; **(B)** Df 27369; **(C)** Df 27372; **(D)** Df 27404; and **(E)** Df 27917, worsened the Aβ42-induced phenotype. **(F)** Df 7681 improved the climbing ability of Aβ42-expressing flies. Df lines had no effect in the absence of Aβ expression. Bars represent the mean ± SEM from at least three independent biological experiments; *p < 0.5, **p < 0.01, ***p < 0.001 (RM one-way ANOVA followed by LSD Fisher’s test).

One hundred and ninety-nine lines with defined deletions from the 2nd, 3rd and 4th chromosomes, accounting for approximately 6300 genes, were tested in the first stage of the screen. Negative geotaxis of G4 > Aβ42 line was compared to G4 > Aβ42/Df lines at 5 and 18 d.p.e. Figure 3 shows actual examples of the three possible outcomes: Df 29667 had no modifying effect, Df 27917 worsened and Df 7681 rescued Aβ42-induced climbing dysfunction. At this stage, 73 G4 > Aβ42/Df lines showed a difference in climbing ability of at least 50% when compared to G4 > Aβ42 and such differences were only seen in aged animals. These lines were selected and analyzed in stage II and six lines met statistical criteria to be considered as positive hits. Df lines 24392, 27369, 27372, 27404 and 27917 worsened negative geotaxis while line 7681 reduced Aβ42 toxicity to a full rescue of the phenotype (Figure 4). In the absence of Aβ42 expression, Df lines showed no intrinsic effect and none of the enhancer Df lines induced climbing impairment in G4 > Aβ42 line at 5 d.p.e, ruling out a possible acceleration of Aβ42 toxicity (not shown). Within these six Df lines, 36 *Drosophila* genes with human orthologs remain to be tested to identify enhancers and 14 genes to pin point suppressors of Aβ42 neurotoxicity. Interestingly, 14 out of 15 enhancer and six out of seven suppressor Df lines described in a previous screen based on negative geotaxis (Liu et al., 2015) were selected in stage I of our screen but did not reach statistical significance in stage II and were not further analyzed.

### Specific Reduction of *CG11796* Expression Enhances Aβ42 Toxicity

**Figure 5:**
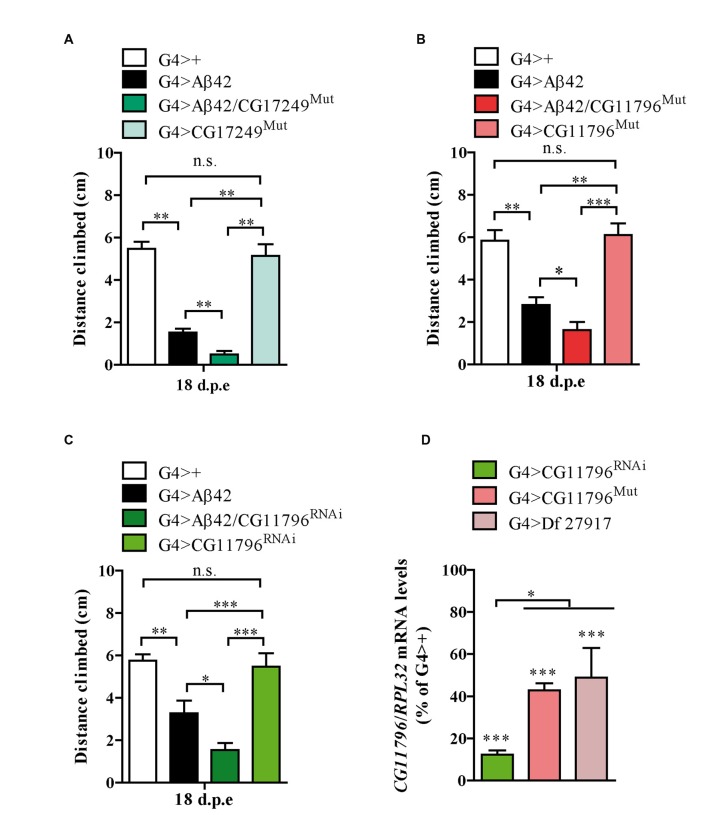
**Mutants and RNAi of candidate genes enhance Aβ toxicity. (A)** *CG17249* hemizygous mutant (human ortholog, *PRCC*); **(B)** *CG11796* hemizygous mutant (human ortholog, *HPD*) and **(C)** *CG11796* RNAi. Mutant and RNAi lines had no effect in the absence of Aβ42 expression. Bars represent mean ± SEM from at least three independent biological experiments; *p < 0.5; **p < 0.01; ***p < 0.001 (one-way ANOVA followed by LSD Fisher’s test). **(D)** Quantification of *CG11796* endogenous mRNA showed a ~40%–50% reduction in Df 27917 and CG11796^Mut^ lines, while for CG11796^RNAi^ a ~ 85% reduction was observed. Brain samples were taken from 5 day-old flies and *RPL32* mRNA was used for normalization in each quantitative real-time PCR (qRT-PCR) assay. ***p < 0.001 for Df 27917, CG11796^Mut^ and CG11796^RNAi^ as compared to G4>+. *p < 0.05 (RM one-way ANOVA followed by LSD Fisher’s test from three independent biological experiments).

Thus far, three out of the five enhancer Df lines that passed stage II have been partially analyzed in stage III. The enhancer Df line 27372 included the deletion of *CG17249* whose human ortholog is *PRCC*. We used a line carrying a Piggy Bac transposon in the 3′ region of *CG17249* to assess toxicity. A significant enhancement in Aβ42 neurotoxicity was observed in mutant hemizygous flies (Figure 5A). Although unlikely, the 3′ insertion may compromise the expression of neighboring genes and therefore, RNAi experiments are required to validate *CG17249*. Df lines 27917 and 27369 also worsened negative geotaxis in the presence of Aβ42 and the overlapping chromosomal segment included *CG11796* whose human ortholog is *HPD* encoding HPPD, a key enzyme involved in tyrosine catabolism. To determine if a reduced expression of *CG11796* was capable of enhancing Aβ42 toxicity, we used two independent approaches: a mutant line in which a Mi[Mic] transposon was inserted in the *CG11796* gene and a specific RNAi with pan-neuronal expression using the *elav* promoter. These lines had no impairment in negative geotaxis as compared with control flies despite the reduction of *CG11796* mRNA. Yet, in the presence of pan-neuronal Aβ42 expression, *CG11796* downregulation in both the RNAi and mutant lines induced a significant enhancement of Aβ42 toxicity, similar to the overall effect of the chromosomal deletions detected at stages I-II of the screen (Figures 5B,C). The specificity of the RNAi was assessed by qRT-PCR from fly heads, which showed a strong reduction of *CG11796* mRNA of approximately 85% in G4 > CG11796^RNAi^ and 55% in CG11796^Mut^, similar to the expected ~50% mRNA reduction in Df line 27917 (Figure 5D).

### Reduction of *CG11796* Expression Promotes the Accumulation of Non-Fibrillar Aβ42

**Figure 6:**
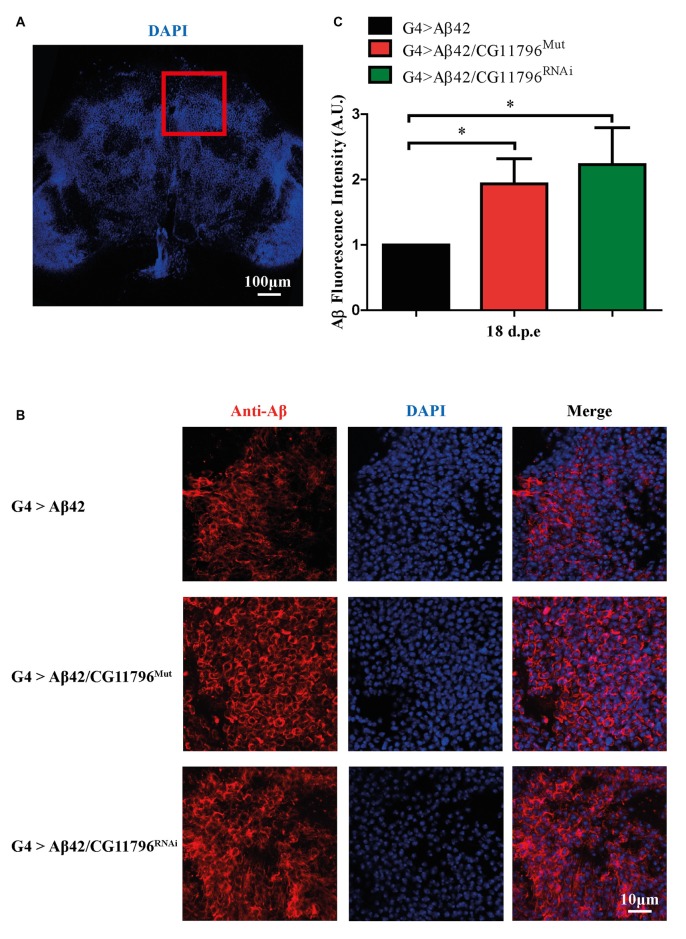
**Immunofluorescence of Aβ deposits in the brains of transgenic lines. (A)** Representative image of a brain section at low magnification stained with DAPI. The red square depicts the region used for quantification in each genotype. Scale bar = 100 μm.** (B)** Representative images of the selected region as in panel **(A)** showing from left to right: anti-Aβ, DAPI nuclear staining and the merge of both signals. Scale bar = 10 μm. Genotypes G4 > Aβ42, G4 > Aβ42/CG11796^Mut^ and G4 > Aβ42/CG11796 ^RNAi^ are shown. **(C)** Quantification of Aβ fluorescence intensity normalized to G4 > Aβ42 in A.U. showing the increment induced by *CG11796* mRNA reduction. Bars represent the mean-ratio ± SEM of three independent experiments; *p < 0.05 (Wilcoxon test).

**Figure 7:**
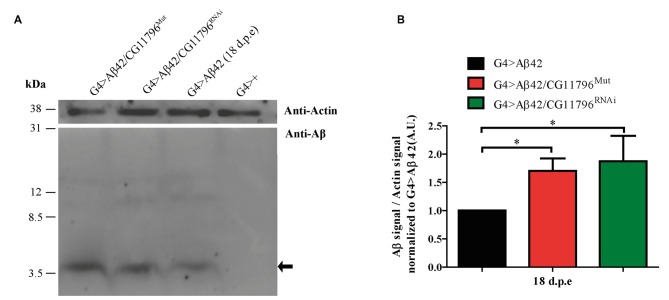
**Western blot of Aβ accumulation in the brain of *CG11796* RNAi and mutant lines. (A)** Representative Western blot of fly brain homogenates in RIPA buffer at 18 d.p.e showing Aβ42 expression detected with monoclonal antibody 6E10. The arrow indicates Aβ42 monomers. Membranes were cut above the 31 kDa marker and probed with anti-actin for normalization. **(B)** Quantification of Aβ42 levels relative to actin in A.U. normalized to G4 > Aβ42 showing the increase of Aβ42 in *CG11796* mutant and RNAi lines. Bars represent the mean-ratio ± SEM of three independent experiments; *p < 0.05 (Wilcoxon test).

**Figure 8:**
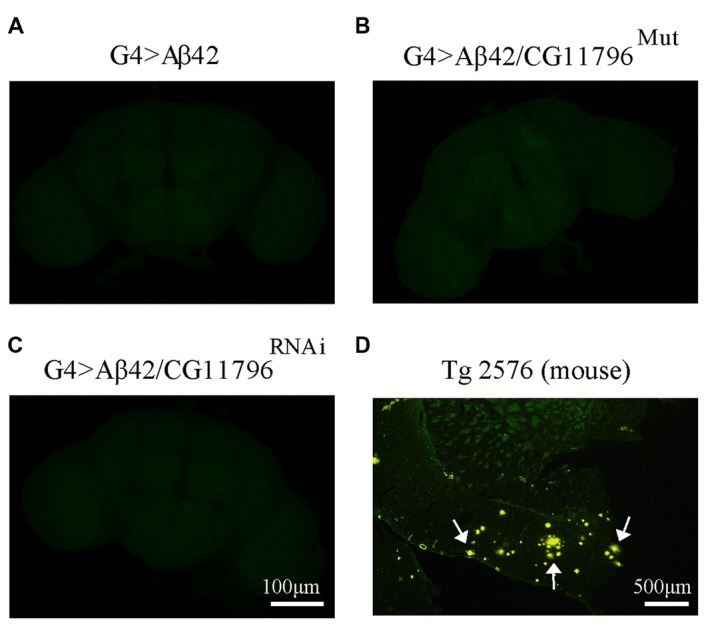
**Negative thioflavine S (ThS) staining of Aβ42 transgenic flies brains. (A–C)** Representative images of ThS staining of fly brains at 18 d.p.e from G4 > Aβ42, G4 > Aβ42/CG11796^Mut^ and G4 > Aβ42/CG11796^RNAi^ showing no detection of amyloid fibrils. Scale bar = 100 μm. **(D)** A brain section of transgenic mouse Tg2576 showing ThS-positive plaques (arrows) is shown for comparison. Scale bar = 500 μm.

**Figure 9:**
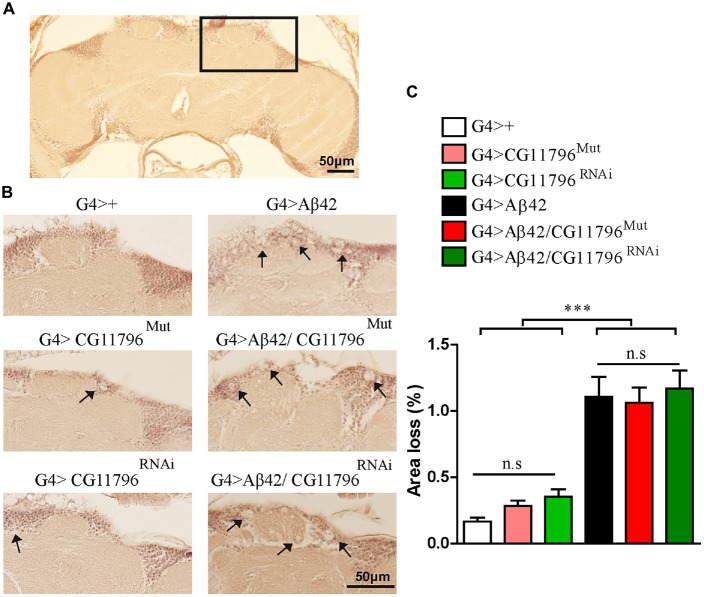
**Brain vacuolization in Aβ-expressing lines alone and in a background of *CG11796* Df. (A)** Representative whole brain section of G4>+ stained with H&E used for tissue loss analysis by bright-field microscopy. The rectangle demarcates a typical area with a high number of neuronal bodies. **(B)** The region depicted in **(A)** is shown for each genotype. Arrows indicate vacuoles with a diameter of at least 3 μm. Scale bar = 50 μm. **(C)** Quantification of tissue loss in hemi-brains was calculated as the percentage of the section area occupied by vacuoles. Flies expressing Aβ42 showed increased vacuolization as compared to control flies G4>+. No differences were found in G4 > Aβ42/CG11796^Mut^ and G4 > Aβ42/CG11796^RNAi^ compared with G4 > Aβ42. ***p < 0.001 (one way ANOVA followed by Tukey’s *post hoc* test).

Aβ42 levels were analyzed in the brains of flies with partial Df of *CG11796* at 18 days of age, when the toxic phenotype was detected. Confocal immunofluorescence showed extensive intraneuronal perinuclear Aβ accumulation which was ~2-fold higher in both *CG11796* hemizygous mutant and CG11796^RNAi^ as compared with flies expressing Aβ42 alone (Figure 6). Western blots of head homogenates showed a 70%–80% increase in the Aβ monomer band in *CG11796* mutant and RNAi lines, consistent with the immunofluorescence results (Figure 7). The increment of Aβ abundance was not accompanied by ThS staining, indicating that a partial Df of *CG11796* expression promoted the accumulation of non-fibrillar Aβ species (Figure 8). Instead, the pattern of immunostaining and detergent solubility suggest the accretion of intraneuronal oligomeric Aβ which concurs with a higher neurotoxicity in CG11796^Mut^ and CG11796^RNAi^ flies. To assess neurodegeneration further, the extent of vacuolization in the brain was determined for each genotype. As mentioned above, there was a mild though significant increase of vacuolization in flies expressing Aβ42 as compared with their genetic controls. Yet, tissue loss did not increase in Aβ42-transgenic flies expressing *CG11796* mutant or RNAi (Figure 9). Together, these results strongly suggest that a partial Df of the HPD ortholog promotes the accumulation of toxic Aβ42 oligomers in the CNS leading to cellular dysfunction without histologically detectable neuronal loss.

## Discussion

The finding of proteins that modulate Aβ neurotoxicity in animals with a complex CNS such as *Drosophila* may impact on AD research in several ways. First, by providing novel players in the cellular mechanisms by which Aβ promotes synaptic dysfunction and neuronal death. Second, changes in the levels or activity of those proteins may be validated in human samples including post-mortem tissue and, more relevant, in biological fluids as potential biomarkers. Third, in the long range, it may open therapeutic strategies alternative to the current ones mostly aimed at Aβ and tau. Previous modifier screens in the fly have yielded interesting candidates that modulate wild-type Aβ toxicity in the eye, upon life span, or negative geotaxis induced by an aggressive Aβ mutant (Cao et al., 2008; Tan et al., 2008; Rival et al., 2009; Liu et al., 2015). Our screen was designed to search for modifiers in a context of neurotoxicity more related to what may occur in sporadic AD, including pan-neuronal expression of wild-type Aβ42 and age-dependent accumulation with no detectable behavioral impairment in young animals. Moreover, the Aβ42 transgenic line had a rather mild phenotype at ~3 weeks of age, with little neuronal loss and the accumulation of detergent-soluble, non-fibrillar species of Aβ, avoiding features that are found in late stages of AD.

The discrepancies between our results and those reported by Liu et al. (2015) may be due to criteria for defining positive hits and the use in their study of AβE22G driven to specific interneurons that relay to thoracic muscles instead of pan-neuronal wild-type Aβ42. Noteworthy, in both studies Df line 7681 was a strong suppressor, suggesting that one or more genes in homozygosity within this deletion are necessary for Aβ to impair geotaxis behavior, independent of Aβ species and type of neurons involved.

A limitation of our study was its restriction to the effect of gene deletions and therefore, likely dependent on lower than physiological levels of the encoded proteins. Those genes that modulate Aβ42 toxicity through overexpression would be missed with our strategy.

So far, two genes have passed stage III of our screen whose human orthologs are *PRCC* and *HPD*. While *PRCC* requires a final validation step with RNAi, *HPD* was unambiguously identified. The function of ppPRCC is largely unknown although early studies suggest that it may have a role in pre-mRNA splicing (Skalsky et al., 2001). A search for ppPRCC protein-protein interactions revealed association with peptidylprolyl isomerase-like 2 (Ppil2), a chaperone with putative ubiquitin ligase activity (Hatakeyama et al., 2001; Pushkarsky et al., 2005; Hegele et al., 2012). Thus, a possible role of a ppPRCC-Ppil-2 complex in protein folding, transport and degradation warrants further study in the context of Aβ neurotoxicity. *HPD* encodes a highly conserved protein that catalyzes the conversion of 4-hydroxyphenylpyruvate to homogentisate, the second step in the tyrosine degradation pathway. Mutations in *HPD* cause the rare diseases Tyrosinemia type 3 and Hawkinsiuria. Tyrosinemia type 3 is autosomal recessive; patients show mental retardation and elevated levels of tyrosine and its derivatives in blood and urine due to HPPD Df (reviewed in Scott, 2006). Hawkinsinuria is autosomal dominant and characterized by metabolic acidosis and urinary excretion of “hawkinsin”, a cyclic amino acid derived from quinolacetic acid produced by mutant HPPD (Brownlee et al., 2010). The mechanisms underlying mental retardation in Tyrosinemia are not known, yet an increase of acetylcholinesterase activity and energy metabolic impairment have been postulated (Ferreira et al., 2012, 2015). In addition, high tyrosine levels may reduce the activity of thiol-dependent creatine kinases (CK) leading to misbalance of a key ATP buffering and shuttling system (Wallimann et al., 2011; de Andrade et al., 2012). Interestingly, CK activity is reduced in AD brains as compared to age-matched controls and Aβ induces a reduction of CK activity in cultured neurons (Aksenov et al., 1998, 2000; David et al., 1998). Consistent with these findings, creatine accumulates in old transgenic mice expressing a mutant APP and in the hippocampus of AD patients (Gallant et al., 2006). Our finding that the partial Df of *HPD* ortholog promoted the accumulation of oligomeric Aβ42 provides a likely explanation for the worsening of age-dependent geotaxis performance. Yet, such degree of Aβ accumulation seems to be sufficient to impact negatively upon neuronal function without inducing gross neuropathological changes up to 18 d.p.e. With regard to possible mechanisms for Aβ accretion in the context of lower HPPD expression, the reduction in CK activity as a consequence of high tyrosine levels may accelerate Aβ aggregation or impair its clearance due to lower ATP availability and oxidative stress (Meyer et al., 2006). Moreover, Aβ42 oligomers induce oxidative stress (Butterfield et al., 2013) leading to a vicious cycle in disease progression. Alternatively, the possibility that a partial Df of HPPD is more directly involved in Aβ accumulation deserves further investigation. Inhibitors of HPPD such as nitisinone are used to treat patients with hereditary Tyrosinemia type 1 in which downstream metabolites of HPPD activity accumulate and are highly toxic to the kidney and liver (Mayorandan et al., 2014; Zeybek et al., 2015). Long-term outcome of patients under nitisinone treatment show a high frequency of progressive cognitive impairment that has been related with chronically elevated tyrosine levels (Masurel-Paulet et al., 2008; Thimm et al., 2012). Early reports on tyrosine levels in the cerebrospinal fluid of AD as compared with controls remain controversial (Degrell et al., 1989; Martinez et al., 1993) and there are no studies on the levels and/or activity of HPPD in AD. In light of our results regarding Aβ accumulation, such studies may be relevant to better understand the complex pathogenesis of AD.

In summary, our work describes the first genetic screen to search for modifiers of wild-type Aβ42 neurotoxicity in the CNS of *Drosophila* by exploring age-dependent alterations in a complex behavior. So far, this strategy has led us to identify candidate genes that warrant further research to determine their significance in sporadic AD.

## Author Contributions

LFB-C, MSM and NIB performed the experiments, analyzed results, drafted and revised the manuscript; MFC and LM designed the work, analyzed data, interpreted the results and revised the manuscript. EMC designed the work, analyzed data, interpreted the results and wrote the article.

## Funding

This work was supported by grants from the Alzheimer’s Association (IIRG 11-205127 to EMC), Agencia Nacional de Promoción Científica y Tecnológica (ANPCyT) PICT2013-0318 (to EMC), PICT2013-1382 (to MFC) and CONICET-PIP0378 (to LM).

## Conflict of Interest Statement

The authors declare that the research was conducted in the absence of any commercial or financial relationships that could be construed as a potential conflict of interest.

## Footnotes

^1^http://www.flystocks.bio.indiana.edu/Browse/df/dfkit.php

^2^http://www.flybase.org

^3^http://www.flyrnai.org/cgi-bin/DRSC_orthologs.pl

^4^http://www.ncbi.nlm.nih.gov/gene

^5^http://flybase.org/reports/FBgn0036992.html

^6^http://www.uniprot.org/

^7^http://www.genecards.org/

^8^http://www.omim.org/

## Abbreviations

AD: Alzheimer’s disease
Aβ: amlyoid β peptide
Aβ42: amyloid β peptide 1-42
CK: creatine kinases
Df: deficiency
DIOPT: *Drosophila* RNAi Screen Center Integrative Ortholog Prediction Tool
dpe: days post-eclosion
G4: Gal4
H&E: hematoxylin-eosin
HPPD: 4-hydroxy-phenylpyruvate dioxygenase
PBS: phosphate buffered saline
Ppil2: peptidylprolyl isomerase-like 2
ppPRCC: proline-rich protein PRCC
qRT-PCR: quantitative real-time PCR
RING: Rapid Iterative Negative Geotaxis
RM: repeated measures
SDS-PAGE: sodium dodecyl sulfate-polyacrylamide gel electrophoresis
ThS: thioflavine S
UAS: upstream activating sequence.
